# A probabilistic framework for clinicopathological Alzheimer’s disease using tau-PET

**DOI:** 10.1186/s13195-026-02133-1

**Published:** 2026-07-11

**Authors:** Bastiaan G J van Tol, Colin Groot, Marie R Vermeiren, Elsmarieke van de Giessen, Yolande A L Pijnenburg, Emma M Coomans, Rik Ossenkoppele

**Affiliations:** 1 Alzheimer Center Amsterdam, Department of Neurology, Vrije Universiteit Amsterdam, Amsterdam UMC location VUmc, Amsterdam, The Netherlands; 2 Neurodegeneration, Amsterdam Neuroscience, Vrije Universiteit Amsterdam, Amsterdam UMC location VUmc, Amsterdam, The Netherlands; 3 Department of Radiology & Nuclear Medicine, Vrije Universiteit Amsterdam, Amsterdam UMC location VUmc, Amsterdam, the Netherlands; 4 Brain Imaging, Amsterdam Neuroscience, Vrije Universiteit, Amsterdam UMC location VUmc, Amsterdam, The Netherlands

**Keywords:** Tau-PET, Alzheimer's disease, biomarkers, [^18^F]flortaucipir

## Abstract

**Background:**

[^18^F]flortaucipir tau-PET detects neurofibrillary tangle (NFT) pathology in Alzheimer’s disease (AD), one of the core pathological hallmarks of the disease. In clinical settings, tau-PET is usually interpreted qualitatively, and therefore quantitative estimates of how scan results alter the probability that AD explains a patient’s symptoms are lacking. Here, we evaluate the probability of clinicopathological AD given a positive or negative tau-PET scan and examine how patient age and amyloid-PET status modulate this probability.

**Methods:**

We computed positive and negative predictive values (PPV/NPV) of tau-PET for clinicopathological AD, defined as mild cognitive impairment or dementia with AD as the primary etiology. To account for potential pathological and clinicopathological mismatches, PPV and NPV were modeled using literature-derived sensitivity and specificity estimates of [^18^F]flortaucipir PET for postmortem Braak V/VI NFT pathology, together with literature-derived age-dependent tau-PET positivity rates in cognitively unimpaired individuals. We also considered hypothetical clinician-estimated pre-PET AD probabilities. PPV and NPV were calculated for tau-PET and for sequential amyloid- and tau-PET scenarios.

**Results:**

Tau-PET PPV for clinicopathological AD was generally high, particularly in individuals with higher clinician-estimated pre-PET AD probabilities, and showed minor age-related declines (e.g., 84% at ages 50–55 vs. 75% at ages 85–90, at 50% pre-PET probability). Tau-PET NPV was consistently higher than PPV and showed negligible age-related decline (e.g., 92% at ages 50–55 vs. 90% at ages 85–90, at 50% pre-PET probability). A positive tau-PET following positive amyloid-PET substantially increased PPV, particularly in older individuals (e.g., PPV increased from 56% to 83% at ages 75–80 and 30% pre-PET probability), whereas the corresponding gain in NPV after tau-PET following amyloid-PET was smaller.

**Conclusions:**

Tau-PET demonstrates high PPV and NPV for clinicopathological AD. A positive tau-PET following positive amyloid-PET further increases the probability of clinicopathological AD, particularly in older adults. This underscores tau-PET’s clinical utility and highlights the effect of age and pre-PET certainty on post-PET AD probabilities.

**Supplementary Information:**

The online version contains supplementary material available at https://doi.org/10.1186/s13195-026-02133-1.

## Background

The diagnostic assessment of Alzheimer’s disease (AD) dementia has advanced substantially with the advent of in vivo PET radiotracers targeting core AD pathologies. Amyloid-PET reliably detects amyloid-β plaques and is widely used to differentiate AD from other causes of cognitive impairment [1]. However, its specificity for symptomatic AD is limited, as incidental or comorbid amyloid-β pathology is common, especially in older adults and in APOE ε4 carriers [2–4]. In contrast, tau-PET radiotracers bind to tau neurofibrillary tangles (NFTs), and demonstrate high sensitivity and specificity for symptomatic AD [5–8], with strong associations to cognitive decline and disease progression. Tau-PET thus offers a key advantage over amyloid-PET by supporting both inclusion and exclusion of AD as the primary etiology of impairment [9–12].

The tau-binding radiotracer [^18^F]flortaucipir demonstrates strong correspondence with advanced neocortical tau pathology, particularly Braak stage V/VI NFT pathology, and has recently received regulatory approval for supporting the diagnosis of AD in cognitively impaired (CI) individuals [13]. While this regulatory milestone facilitates clinical implementation, several important caveats remain. First, pathological mismatches may occur due to false-positives (e.g., elevated [^18^F]flortaucipir uptake due to off-target binding) or false-negatives (e.g., subthreshold tracer uptake despite the presence of Braak V/VI NFT pathology) [14]. Second, although relatively uncommon, tau-PET positivity is occasionally observed in individuals without cognitive impairment, with prevalence increasing with age, resulting in age-dependent clinicopathological mismatches [11, 15, 16]. Third, tau-PET findings require interpretation within a broader clinical context, including demographic factors, clinical presentation, family history, and complementary biomarkers such as amyloid-β status [1]. Therefore, accounting for these factors is essential for an accurate and clinically meaningful interpretation of tau-PET.

Here, we aimed to determine the positive predictive value (PPV) and negative predictive value (NPV) of tau-PET for clinicopathological AD, defined as the presence of mild cognitive impairment (MCI) or dementia clinical syndromes that are primarily attributable to underlying AD pathology, characterized by the co-occurrence of both amyloid-β and advanced neocortical tau pathology (Braak stage V/VI NFT). Building on prior work on amyloid-PET [17], we applied a probabilistic framework to estimate how tau-PET results, alone and in sequential combination with amyloid-PET, update the probability of AD across clinical scenarios. We hypothesized that tau-PET negativity would yield a particularly high NPV, supporting the exclusion of clinicopathological AD, and that tau-PET positivity would also provide a high, albeit lower, PPV for identifying AD as the primary etiology of symptoms. Finally, we illustrate clinical applicability using two illustrative cases from the TAP-TAU study [18].

## Materials and methods

### Computation of positive and negative predictive value

PPV and NPV were defined using standard formulas:

$$\:PPV\:=\:\frac{True\:Positives}{True\:Positives\:+\:False\:Positives}$$

$$\:NPV\:=\:\frac{True\:Negatives}{True\:Negatives\:+\:False\:Negatives}$$

To compute PPV and NPV, we adapted our previously published amyloid-PET framework [17] by integrating: (i) sensitivity and specificity of ante-mortem [^18^F]flortaucipir PET for post-mortem Braak stage V/VI NFT pathology, (ii) age-dependent tau-PET positivity estimates in cognitively unimpaired (CU) individuals [16], and (iii) a broad range of hypothetical clinician-estimated pre-PET probabilities of AD.

### Model inputs

#### Pre-PET probability of Alzheimer’s disease

We incorporated clinician-estimated pre-PET probabilities of AD, representing the clinician’s judgement of the probability that a patient’s cognitive symptoms are primarily attributable to AD, given all available clinical information prior to tau-PET imaging. This pre-PET probability reflects a subjective probabilistic estimate, analogous to the pre-test probability in standard Bayesian diagnostic reasoning. We modeled a range of hypothetical values, from 10% to 90% (with 10% increments), to reflect varying levels of diagnostic (un)certainty.

#### Tau-PET correspondence with postmortem tau pathology

To account for the diagnostic accuracy of tau-PET in detecting advanced tau pathology, we incorporated literature-derived sensitivity and specificity estimates for identifying Braak stage V-VI NFT pathology at autopsy. Analyses were limited to [^18^F]flortaucipir, as ante-mortem PET vs. post-mortem neuropathological data currently only exist at sufficiently large scale for this tracer. We conducted a literature review of cohort studies published between January 2013 and February 2026 that directly compared ante-mortem temporal [^18^F]flortaucipir PET standardized uptake value ratios (SUVR) with post-mortem Braak V/VI NFT staging in the same individuals, using the following PubMed search terms: (flortaucipir OR AV1451 OR TAUVID) AND (post-mortem OR autopsy OR neuropath*).

From each eligible study, we extracted sample size, regions of interest (ROIs), tau-PET cutoff approaches, and diagnostic performance metrics. These data were complemented with additional analyses in the Alzheimer’s Disease Neuroimaging Initiative (ADNI) and the Amsterdam Dementia Cohort (ADC), using the same methodology (Youden’s index to identify Braak V/VI NFT pathology) as in our prior work [9]. Primary analyses focused on the AD-specific temporal meta-ROI [19]. When the temporal meta-ROI was unavailable, we selected the closest available neocortical ROI from each respective study. This resulted in selection of the MUBADA-neocortex composite region for Fleisher et al. [13] and the inferior temporal cortex for Josephs et al. [20]. While these regions show substantial spatial overlap with the temporal meta-ROI in capturing advanced neocortical tau pathology, the MUBADA-neocortex additionally encompasses posterior and frontal voxels beyond the temporal meta-ROI, whereas the inferior temporal cortex represents a single ROI rather than a composite ROI.

Eight studies met the inclusion criteria [9, 13, 20–24]. After excluding overlapping subsets [21–23], five independent datasets contributed to pooled estimates [9, 13, 20, 24]. Pooled sensitivity and specificity were computed directly as ΣTP/(ΣTP + ΣFN) and ΣTN/(ΣTN + ΣFP) across all five studies. Contingency counts were obtained by three approaches: For Josephs et al. [20], counts were derived algebraically from the author-provided sensitivity, specificity, and Braak V/VI and Braak < V sample sizes (details in Table-1, footnote b); for Fleisher et al. [13] and Vrillon et al. [24], counts were directly extracted from published tau-PET SUVR by Braak stage figures; and for ADNI and ADC data [9], counts were derived from in-house analyses of individual-level data (Supplementary Figs. 1–2). These contingency counts yielded a pooled sensitivity of 92.7% and specificity of 84.2% for temporal tau-PET in detecting Braak V/VI pathology (Table-1), which were then used as fixed model inputs in the probabilistic model.

**Table 1:** Overview of included studies reporting *temporal* tau-PET sensitivity and specificity for Braak V/VI tau pathology

Study	Sample size (*N*)	Age at PET	CU / MCI / Dem (%)	PET-Autopsy interval	ROI used	TP	FN	TN	FP	SEN (%)	SPE (%)
Josephs et al. [20] ^a, b^	248	75 years (median)	17 / 10 / 72	2.2 years (median)	Inferior temporal	100	3	113	32	97.1	78.0
Vrillon et al. [24]	71	67 years (median)	0 / 0 / 100	3.9 years (median)	Temporal meta	38	2	31	0	95.0	100
Fleisher et al. [13]	60	83 years (mean)	22 / 2 / 77	2.6 months (mean)	MUBADA neocortex	32	6	22	0	84.2	100
ADNI [9] ^c^	12	83 years (median)	17 / 33 / 50	4.2 years (median)	Temporal meta	3	1	7	1	75.0	87.5
ADC [9] ^c^	9	70 years (median)	0 / 0 / 100	2.5 years (median)	Temporal meta	4	2	3	0	66.7	100
Pooled	400					177	14	176	33	92.7	84.2

^a^For the study of Josephs et al. [20], the sensitivity and specificity data were kindly provided by the authors upon request as they could not be distilled from the original publication.

^b^Contingency counts (TP, FN, TN, FP) were derived algebraically from the author-provided sensitivity, specificity, and Braak V/VI positive and negative sample sizes as follows: TP = sensitivity × N Braak V/VI, FN = (1 − sensitivity) × N Braak V/VI, TN = specificity × N Braak

^c^For the ADNI and ADC datasets from Coomans et al. [9], the authors provided the underlying data, enabling calculation of sensitivity and specificity metrics.

Additional details on included studies, including ROI definitions, thresholding approaches, and sensitivity and specificity estimates for whole-brain tau-PET, are provided in Supplementary Tables 1–2.

## Tau-PET positivity in cognitively unimpaired individuals

To account for age-related false positives and false negatives, we incorporated age-dependent prevalence estimates of tau-PET positivity in CU individuals, based on a large multicenter cohort study of 7,394 CU individuals aged 50–90 years [16] (Supplementary Table 3). Within this framework, tau-PET positivity in CU individuals either reflects pathological mismatch (tau-PET positivity in the absence of Braak V/VI NFT pathology) or clinicopathological mismatch, i.e., advanced tau pathology is present but is not the primary driver of symptoms.

### Model implementation

**Fig. 1:**
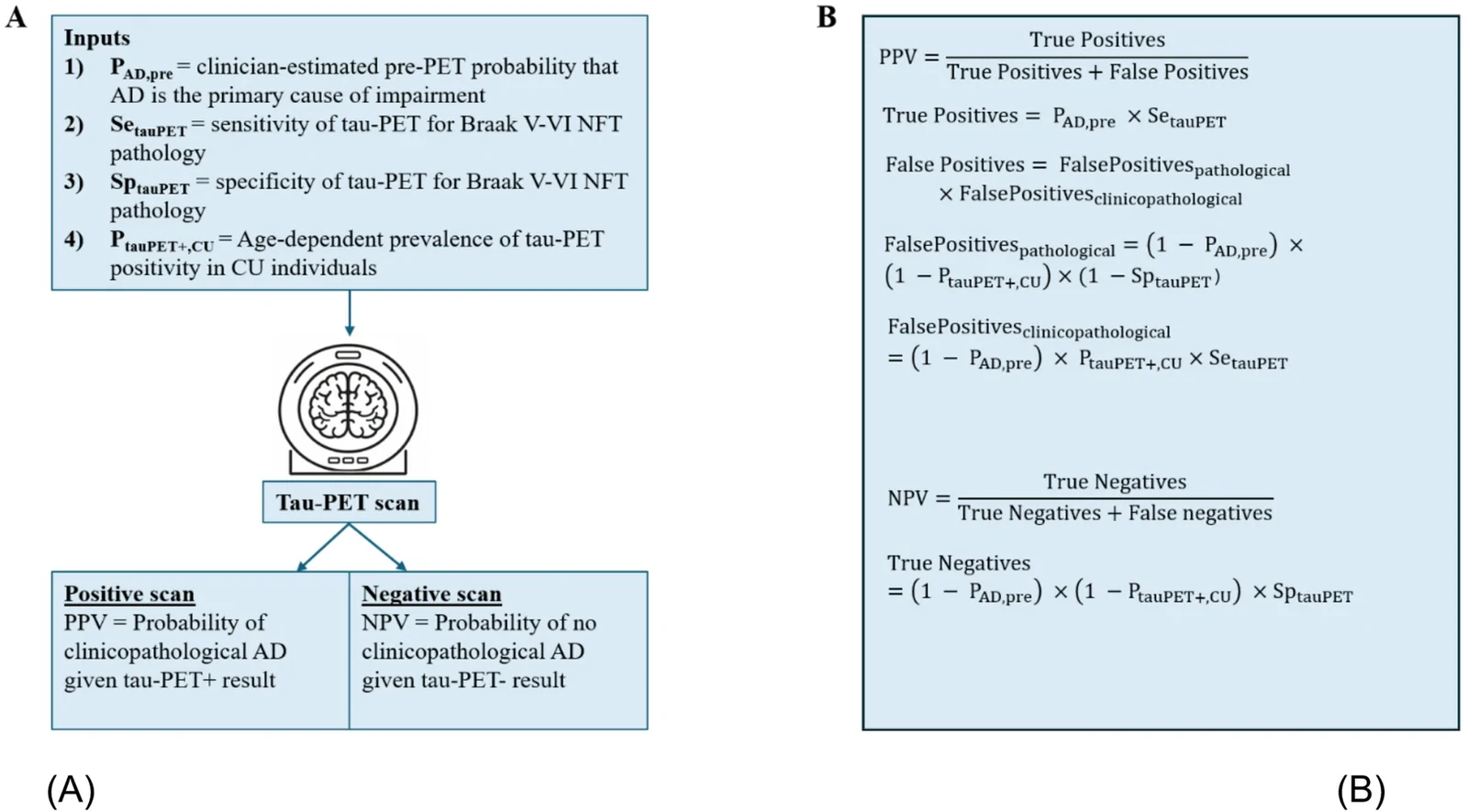
Probabilistic framework for computing PPV and NPV of tau-PET for clinicopathological AD. **A **Panel illustrating the model inputs and probability-updating workflow following a positive or negative tau-PET result. **B** Panel shows the equations to compute PPV and NPV

Tau-PET PPV and NPV for clinicopathological AD were computed across clinician-estimated pre-PET AD probabilities (10–90%) and age groups (50–90 years, five-year intervals) using tau-PET sensitivity, specificity, and age-dependent tau-positivity rates in CU individuals. Results were summarized in two-dimensional tables, where PPV represents the probability of clinicopathological AD given a positive tau-PET scan, and NPV represents the probability of not having clinicopathological AD given a negative scan. Notably, the model makes no assumptions about the frequency of tau-PET positivity at any given pre-PET probability level; it merely quantifies the diagnostic probability update, which is conditional on an observed scan result. Pre-PET probabilities of 0% and 100% were excluded to reflect clinically realistic levels of diagnostic uncertainty. The 5-year age bins were based on published tau-PET positivity prevalence data [16], with minor adjustments on the age-bracket boundaries (Supplementary Table 3). Model structure and equations are shown in Fig. 1, and definitions are provided in more detail in Supplementary Table 4. All analyses were performed in Python (version 3.10), using standard scientific libraries, including NumPy, pandas, matplotlib, and seaborn. All code and summary data are publicly available (OssenKoppeLab/BvanTol_TauPET_PPV_NPV).

To quantify the degree of uncertainty in the pooled sensitivity and specificity estimates, we implemented a bootstrap procedure with 5,000 iterations, from which 95% confidence intervals were derived for all PPV and NPV values (see details on the bootstrap procedure in the legend of Supplementary Table 5). Point-estimate PPV and NPV values reported in the Results section are accompanied by their corresponding 95% CIs from this bootstrap analysis.

### Combined amyloid- and tau-PET modeling

To assess the impact of sequential biomarker testing on post-PET probabilities of clinicopathological AD, we extended the model to scenarios combining amyloid- and tau-PET. In the *amyloid-first* approach, amyloid-PET PPV and NPV for clinicopathological AD were first computed using published prevalence estimates of amyloid-PET positivity in CU individuals [2] (Supplementary Table 7), together with autopsy-validated amyloid-PET sensitivity (92%) and specificity (97%) for detecting amyloid-β pathology [17]. The resulting PPV (representing AD probability given an amyloid-positive scan) and 1–NPV (representing AD probability given an amyloid-negative scan) were then used as updated pre-PET probabilities for subsequent tau-PET modeling. In this way, the model quantified how the probability of clinicopathological AD changes after obtaining a tau-PET scan when amyloid-PET status is already known.

We additionally evaluated a *tau-first scenario*, in which tau-PET preceded amyloid-PET. This analysis was treated as secondary because such a diagnostic sequence is relatively uncommon in routine clinical practice. In this scenario, tau-PET PPV and 1–NPV values were used as the updated pre-PET probabilities when computing amyloid-PET PPV and NPV, thereby modeling the diagnostic impact of amyloid-PET following an initial tau-PET result.

For both sequences, results were summarized in tables analogous to the standalone tau-PET results, presenting PPV and NPV estimates across a range of pre-PET probabilities and age groups between 50 and 90 years (five-year intervals).

## Illustrative clinical case descriptions

Two illustrative cases were selected from the multicenter Dutch TAP-TAU study [18], which enrolls individuals with substantial diagnostic uncertainty regarding AD (defined as clinician-reported confidence of < 85% for AD as the most likely etiology). Participants in this study undergo tau-PET imaging as part of a comprehensive clinical work-up aimed at evaluating the added clinical value of tau-PET in diagnostically challenging cases. This work-up includes clinical history, neuropsychological assessment, structural MRI, and, when available, additional biomarker information such as amyloid-PET or cerebrospinal fluid analyses. Tau-PET scans were visually interpreted by an expert reader (EvdG) who was blinded to the clinical information, following FDA-approved interpretation guidelines [13]. After disclosure of the tau-PET results, clinicians revised their diagnostic certainty regarding AD without access to the PPV/NPV tables generated by the probabilistic model. The revised clinician-estimated probabilities of AD were then compared with the corresponding model-derived PPV and NPV values to illustrate the clinical interpretability and potential practical application of the probabilistic outputs.

## Results

### Positive predictive value and negative predictive value of tau-PET

**Fig. 2:**
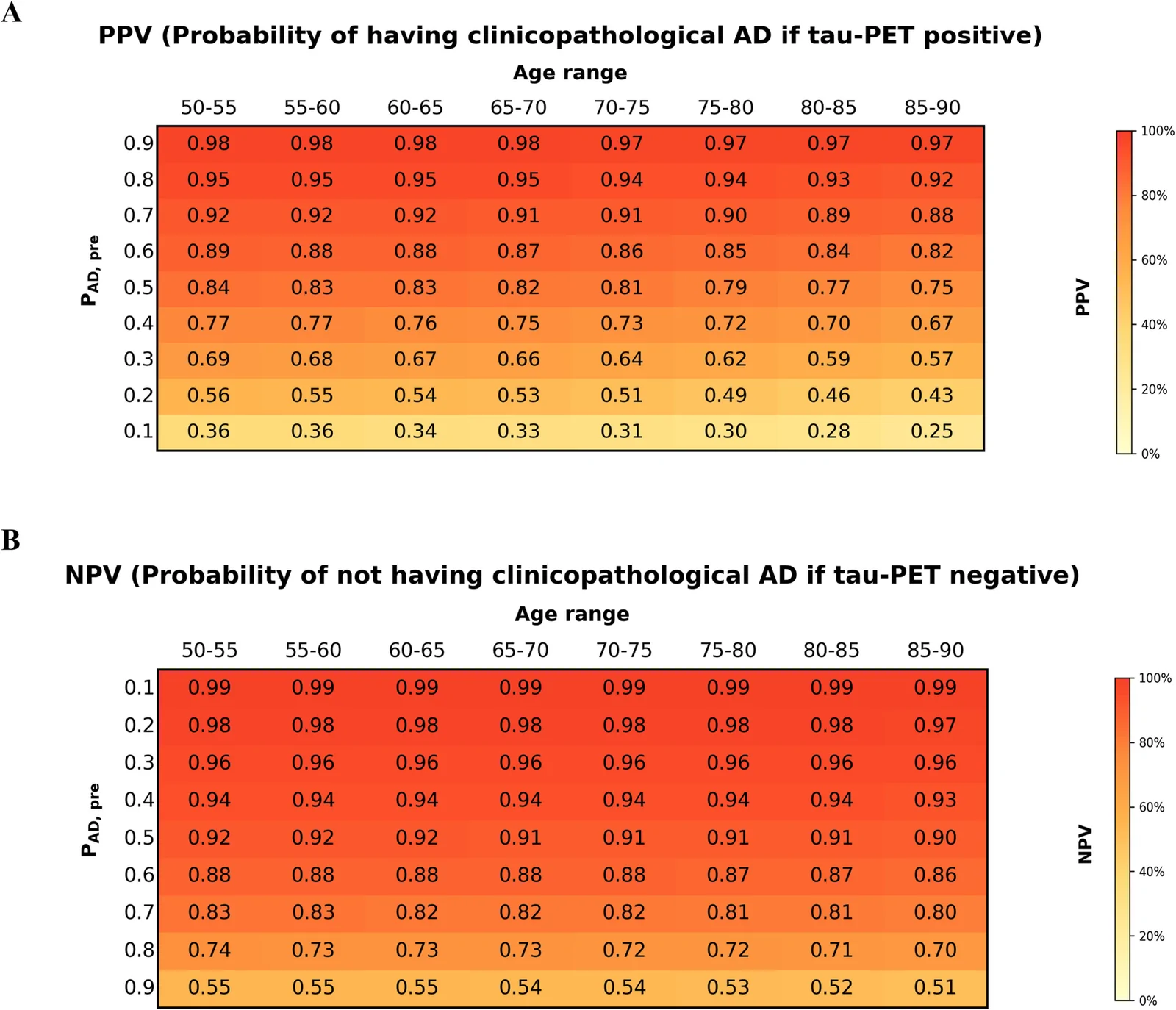
Positive and negative predictive value of standalone tau-PET. Heatmaps showing the probability of (**A**) having clinicopathological AD given a positive tau-PET and (**B**) not having clinicopathological AD given a negative tau-PET, across age groups (X-axis) and clinician-estimated pre-PET probabilities of AD (Y-axis). Each cell represents the diagnostic value of tau-PET in a given clinical context, with more intense colors indicating higher post-PET probabilities

Overall, we observed high PPVs of tau-PET for clinicopathological AD, and across the range of modeled scenarios. Across all age groups, PPV consistently exceeded the P_AD,pre_ values, indicating that a positive tau-PET scan systematically increased the estimated probability of underlying AD (Fig. 2A). Although PPV remained high overall, a modest decline was observed with increasing age. For example, at a pre-PET AD probability of 50%, PPV decreased from 84% [80% − 87%] at ages 50–55 to 75% [73% − 78%] at ages 85–90. This age-related reduction was most pronounced at lower pre-PET AD probabilities, whereas at higher pre-PET probabilities, the effect of age on PPV was minimal. The largest increases in AD probability were observed in individuals with relatively low pre-PET probabilities. For example, at ages 60–65, a positive tau-PET increased the probability of clinicopathological AD from a pre-PET probability of 30% to 67% [62% − 73%] post-PET, corresponding to a substantial upward revision of AD likelihood. In contrast, when the pre-PET probability was already relatively high (e.g., 70%), the same positive tau-PET scan increased the probability to a PPV of 92% [90% − 94%], representing a smaller but still meaningful increase in diagnostic certainty.

NPV values were uniformly high and consistently exceeded the corresponding PPV values, underscoring the strong ability of tau-PET to exclude clinicopathological AD when the scan is negative (Fig. 2B). Age-related variation in NPV values was even more limited than that observed for PPV. Only small reductions with advancing age were observed, primarily at higher pre-PET AD probabilities. For example, at a pre-PET probability of 80%, NPV decreased slightly from 74% [65% − 84%] at ages 50–55 to 70% [60% − 81%] at ages 85–90, indicating that the negative predictive value of tau-PET remained largely stable across the age spectrum. NPV was highest among individuals with lower pre-PET AD probabilities. For instance, at ages 60–65, a negative tau-PET scan yielded an NPV of 96% [94% − 98%] when the pre-PET probability was 30%. By comparison, when the pre-PET probability was higher (70%), the corresponding NPV decreased to 82% [75% − 89%].

Bootstrapped 95% confidence intervals for all PPV and NPV values are provided in Supplementary Tables 5–6. Overall, CIs were relatively narrow, particularly at higher pre-PET AD probabilities, supporting the robustness of the provided point estimates in the main analyses. However, CIs expanded at lower pre-PET probabilities, potentially reflecting the greater influence of sampling variability in the pooled sensitivity and specificity estimates in this range.

Supplementary Fig. 3 displays PPV and NPV tables for temporal meta-ROI tau-PET stratified by APOE ε4 status. For PPV, values were highest in ε4 non-carriers and decreased progressively in heterozygous and homozygous carriers, with more pronounced reductions at older ages and lower pre-PET probabilities. NPV showed a similar pattern across all APOE groups at low pre-PET probabilities but decreased more markedly with advancing age and higher pre-PET probabilities in ε4 carriers compared to non-carriers.

Supplementary Fig. 4 displays the PPV and NPV tables for whole-brain tau-PET, which showed patterns broadly similar to those observed for temporal tau-PET. However, the resulting PPV and NPV values were modestly lower overall and exhibited slightly reduced age-related variation compared with the temporal tau-PET.

### Positive and negative predictive values of amyloid-PET and subsequent tau-PET

**Fig. 3:**
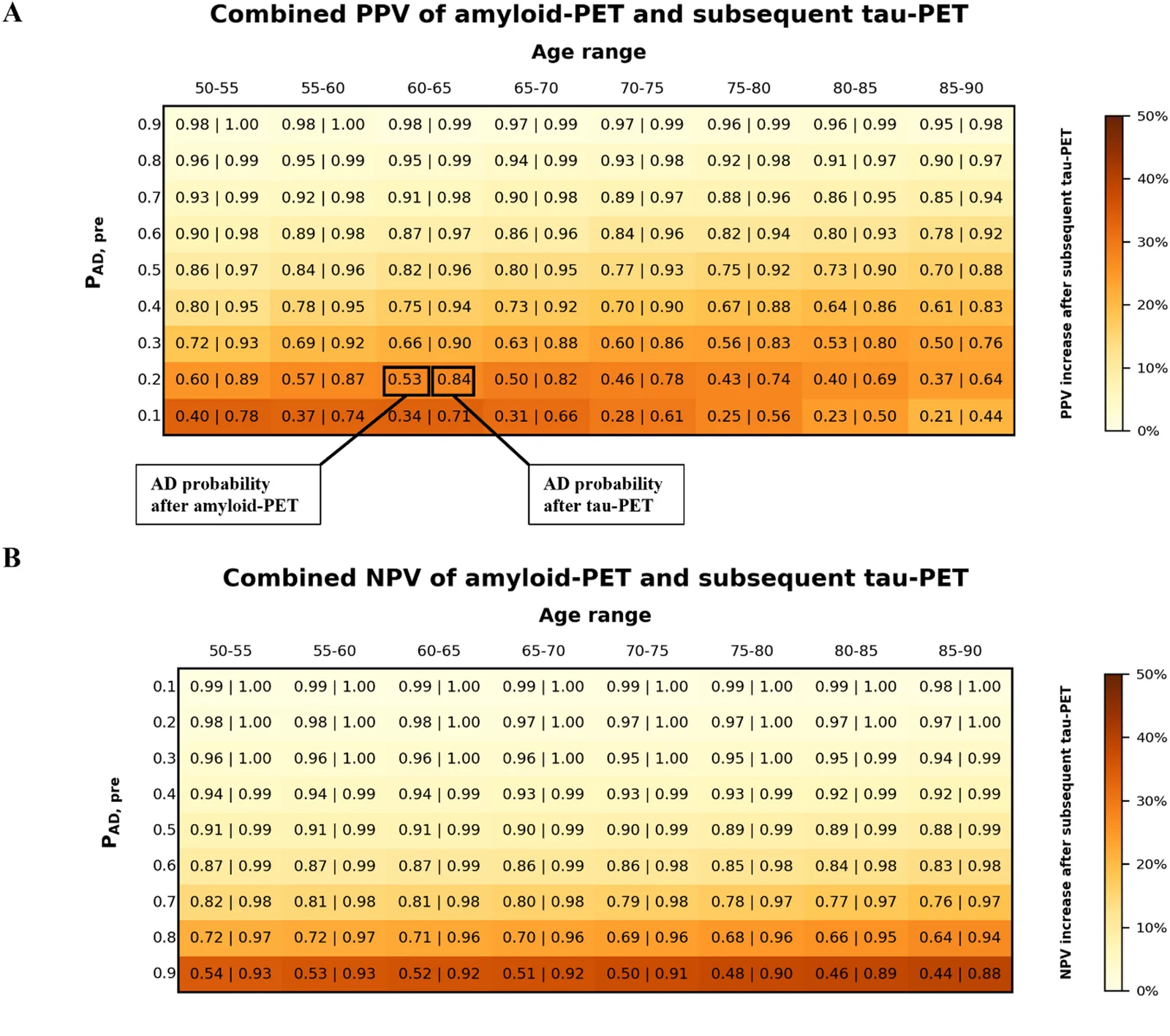
Positive and negative predictive values of amyloid-PET and subsequent tau-PET. Panel **A **shows PPVs (probability of having clinicopathological AD) after a positive amyloid-PET and subsequent positive tau-PET. Panel **B **shows NPVs (probability of not having clinicopathological AD) after a negative amyloid-PET and subsequent negative tau-PET. Each cell presents two values: the predictive value after amyloid-PET alone (first value) and after both scans (second value). The color gradient reflects the change in predictive value, highlighting the added diagnostic gain from tau-PET

Next, we examined PPV and NPV for clinicopathological AD in scenarios involving sequential amyloid-PET and tau-PET testing. In the *amyloid-first* scenario, PPV increased substantially when a positive tau-PET result followed a positive amyloid-PET scan, compared with the PPV obtained after standalone amyloid-PET (Fig. 3A). This gain in AD probability was most pronounced among individuals with lower pre-amyloid-PET probabilities. For example, at ages 60–65 and a pre-amyloid-PET AD probability of 30%, a positive amyloid-PET yielded a PPV of 66%, which increased to 90% after a subsequent positive tau-PET (ΔPPV = + 24%). The additional diagnostic value of tau-PET following amyloid-PET also increased with advancing age. At a pre-amyloid-PET probability of 60%, a positive tau-PET increased PPV by 8% at ages 50–55 (from 90% to 98%), whereas at ages 85–90 the increase in PPV was larger, rising by 14% (from 78% to 92%).

The NPV pattern for clinicopathological AD in the combined *amyloid-first* scenario resembled the pattern observed for standalone tau-PET, with the highest NPVs found among individuals with lower pre-amyloid-PET AD probabilities (Fig. 3B). The largest increases in NPV following tau-PET (relative to NPV after standalone amyloid-PET) were observed, however, in individuals with higher pre-amyloid-PET AD probabilities, indicating that a negative tau-PET scan provided the greatest additional value for ruling out AD in cases where baseline probability of AD was relatively high.

### Positive and negative predictive values of tau-PET with subsequent amyloid-PET

**Fig. 4:**
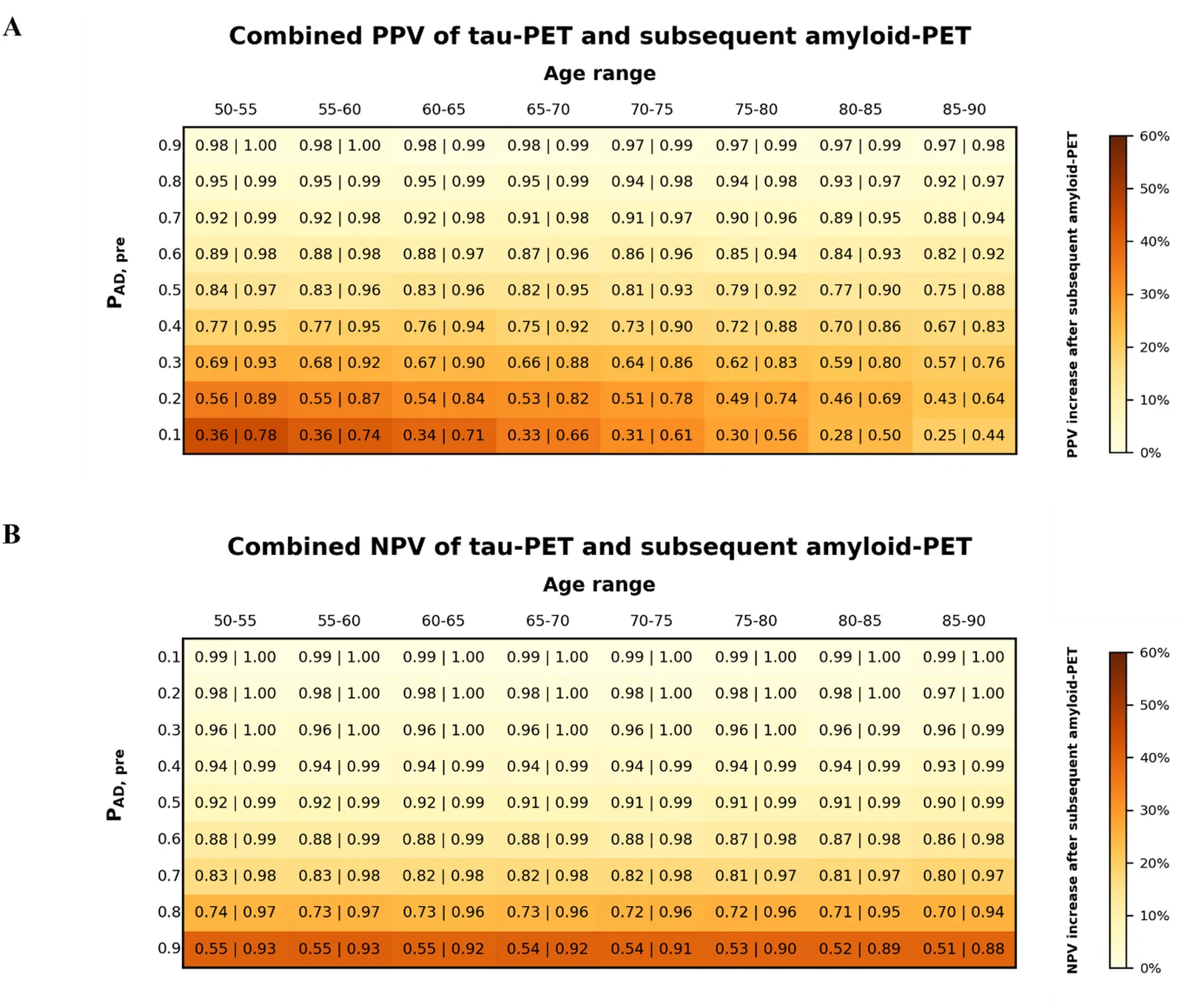
Positive and negative predictive values of tau-PET and subsequent amyloid-PET. Panel **A** shows PPVs (probability of having clinicopathological AD) after a positive tau-PET and subsequent positive amyloid-PET. Panel **B** shows NPVs (probability of not having clinicopathological AD) after a negative tau-PET and subsequent negative amyloid-PET

We next evaluated the *tau-first* scenario, in which amyloid-PET would be obtained after a tau-PET scan. Compared with the *amyloid-first* scenario (where tau-PET was the second scan), the incremental effect of amyloid-PET on PPV after a positive tau-PET result was markedly smaller, particularly at older age. For example, at ages 80–85 and a pre-PET AD probability of 30%, PPV increased from 59% (post tau-PET) to 80% after subsequent amyloid-PET (ΔPPV = + 21%) (Fig. 4A). Under the same conditions, the reverse testing sequence (amyloid-PET first) yielded a larger PPV increase after tau-PET (+ 27%; Fig. 3A).

The NPV pattern in the *tau-first* scenario was broadly similar to that of the *amyloid-first* scenario, with the highest gains in NPV occurring among individuals with low pre-PET AD probabilities (Fig. 4B). However, the increases in NPV following the second scan were generally smaller in the tau-first sequence, and diminished more rapidly as pre-PET AD probabilities decreased, indicating a more limited additional contribution of amyloid-PET in this testing order.

Supplementary Figs. 5–6 present the *amyloid-first* and *tau-first* PPV and NPV tables for amyloid-PET and whole-brain tau-PET. Overall patterns were comparable to those observed for temporal tau-PET, although gains in PPV and NPV after the second scan were smaller, consistent with the slightly lower standalone performance of whole-brain tau-PET (Supplementary Fig. 4).

### Illustrative clinical tau-PET cases

**Fig. 5:**
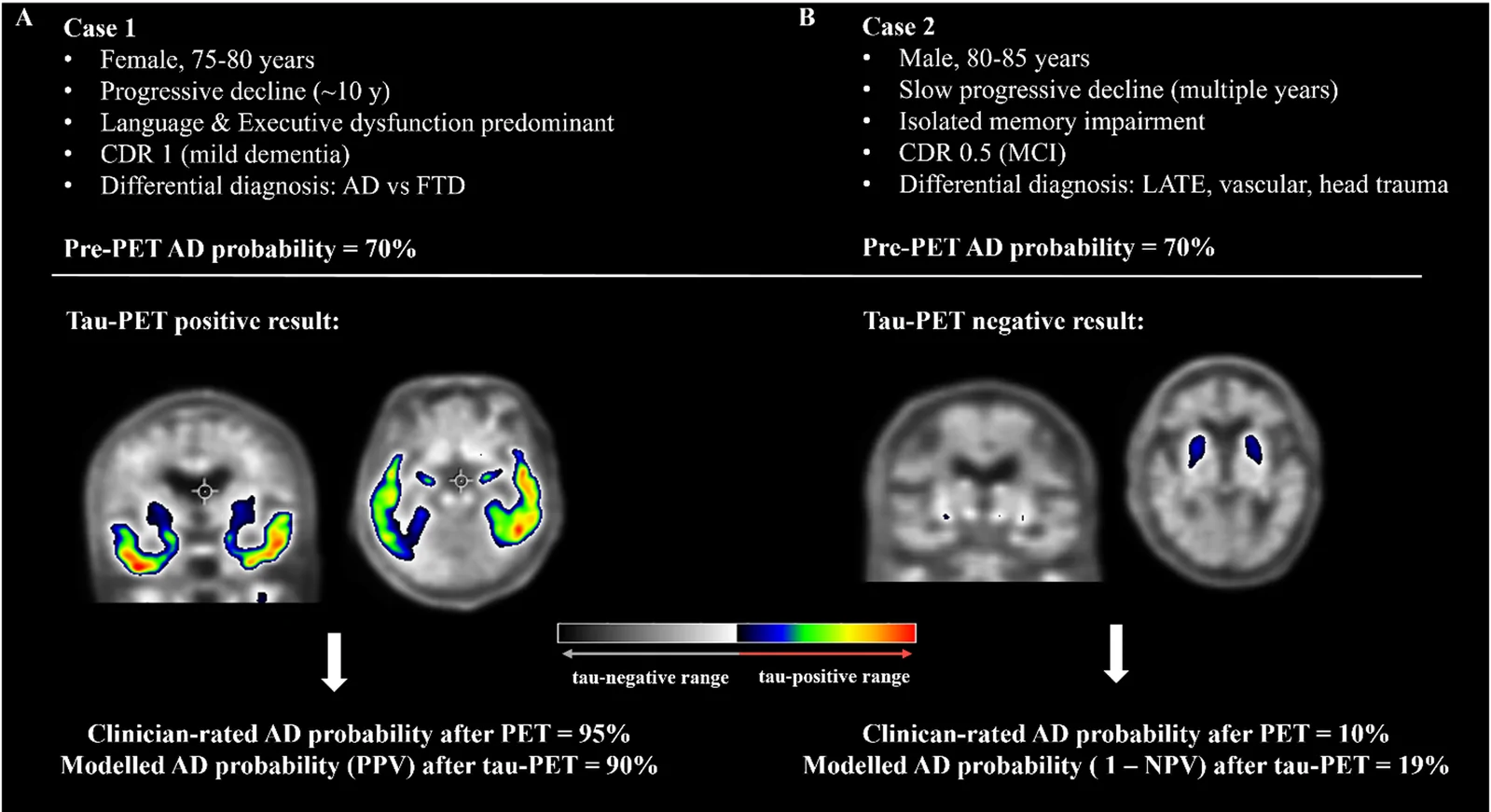
Illustrative tau-PET cases from the TAP-TAU cohort study demonstrating updating of AD probabilities. Panel **A **depicts a patient with a positive [^18^F]flortaucipir scan showing elevated neocortical tau uptake consistent with advanced AD-related NFT pathology [13]. Panel **B **depicts a patient with a visually negative tau-PET scan, showing only non-specific subcortical binding

Two illustrative cases from the TAP-TAU study [18] are shown in Fig. 5 to demonstrate the clinical interpretability of the probabilistic model. In both cases, clinicians estimated a pre-PET probability of AD of 70% following routine clinical evaluation. In the tau-PET-positive case (Fig. 5A**)**, a woman in her mid-seventies with progressive cognitive decline showed a tau-PET pattern consistent with AD-related tau pathology. The modeled probability of clinicopathological AD was 90% (PPV). In the tau-PET-negative case (Fig. 5B), an older male with slowly progressing memory complaints showed no evidence of AD-related tau deposition on tau-PET. The modeled probability of AD was 19% (1 − NPV), while the clinician revised the probability of AD to 10%. Overall, model-derived post-PET probabilities of AD closely aligned with clinician-estimated diagnostic probabilities in both cases, though the difference was slightly larger in the negative tau-PET case compared to the positive tau-PET case.

## Discussion

In this study, we quantified the PPV and NPV of [^18^F]flortaucipir PET for clinicopathological AD using a probabilistic framework that integrates tau-PET sensitivity and specificity, tau-PET positivity prevalences in CU individuals, and clinician-estimated pre-PET AD probability. Tau-PET demonstrated strong performance across a wide range of scenarios, with PPV highest at high pre-PET probabilities, whereas NPV was consistently high at low pre-PET probabilities. When tau-PET was obtained after amyloid-PET, both PPV and NPV increased further, particularly in older individuals and in those with low initial AD probability, with greater gains than in the reverse (*tau-first*) sequence. These findings illustrate how tau-PET can meaningfully refine diagnostic probabilities, particularly in clinically ambiguous cases.

Our findings align with the updated appropriate use criteria for amyloid- and tau-PET, which emphasize the value of tau-PET in situations of substantial diagnostic uncertainty [1]. Consistent with this framework, we observed the largest increases in AD probability after a positive tau-PET in individuals with low pre-PET AD probabilities, where new biomarker information carries the greatest diagnostic weight. In parallel, tau-PET showed strong exclusion power, reflected by consistently high NPV values that exceeded PPV. The minor age-related reductions in PPV at lower pre-PET probabilities likely reflect increasing tau-PET positivity in CU individuals [16] due to non-specific tracer binding, early-stage tau pathology not yet clinically manifest, or comorbid tau pathology in non-AD dementias [7, 11, 25, 26]. Interestingly, supplemental analyses using whole-brain tau-PET showed directionally similar effects, but lower PPV and NPV values compared to the temporal meta-ROI, reflecting its slightly reduced sensitivity and specificity for advanced Braak V/VI pathology. Together, these results illustrate how age and pre-test AD probability jointly shape the clinical interpretation of tau-PET results.

The APOE ε4-stratified analyses revealed progressively lower PPV and NPV values with increasing ε4 dosage, consistent with the substantially higher age-dependent tau-PET positivity rates in CU ε4 carriers [16]. APOE ε4 is associated with accelerated amyloid-β accumulation, which in turn drives earlier and more widespread neocortical tau deposition [27]. This higher tau-PET positivity in ε4 carriers means that a greater proportion of CU ε4 carriers test positive without meeting our definition of clinicopathological AD, thereby reducing the PPV and NPV. These findings suggest that APOE status, when available, may be a relevant consideration when interpreting tau-PET results, particularly in older ε4 homozygous individuals where tau-PET positivity in the reference population is highest. Nonetheless, across all APOE groups, a positive tau-PET scan still substantially increased the probability of clinicopathological AD compared to pre-PET estimates, underscoring its continued clinical utility. Sex-stratified analyses were not performed, as the relevant tau-PET positivity prevalence estimates were not publicly available. However, given that female sex has been associated with higher tau-PET positivity in CU individuals [16, 28], extending this framework to incorporate sex-stratified prevalence data represents an important avenue for future work.

The diagnostic value of tau-PET relative to amyloid-PET in sequential PET testing was dependent on age and scan order. In younger individuals, both modalities performed similarly, yielding high PPV and NPV, whereas at older age, tau-PET outperformed amyloid-PET due to smaller age-related declines in PPV. This difference likely reflects the steeper age-related rise in amyloid positivity, even in CU individuals (~ 25% at age 70), compared to the more modest increase in tau positivity (~ 7.5% at age 70, and only ~ 2% among amyloid-negative individuals) [2, 16]. Accordingly, tau-PET, when performed after amyloid-PET, particularly increased probabilities in older individuals with low to intermediate pre-PET probabilities, whereas amyloid-PET following tau-PET yielded limited additional gains. These findings support using tau-PET after amyloid-PET in diagnostically challenging older patients and suggest that, if only one scan is feasible, tau-PET may offer greater utility in this group. Looking ahead, fluid-based biomarkers such as ptau-217 have emerged as promising and more accessible alternatives to amyloid-PET for detecting AD pathology [29, 30]. Extending the sequential testing framework presented here to incorporate ptau-217 as a first-line biomarker, as a replacement for amyloid-PET, represents a clinically meaningful direction for future work, given its lower patient burden and wider accessibility compared to PET imaging.

Our findings complement earlier studies assessing the clinical utility of amyloid- and tau-PET based on diagnostic changes and clinician confidence [5, 31]. Whereas these studies emphasize subjective diagnostic confidence, our work provides quantitative, data-driven estimates of diagnostic value using PPV and NPV. In contrast to Altomare et al. [5], who reported similar utility irrespective of scan order, our results indicate that tau-PET adds greater diagnostic value when performed after amyloid-PET, particularly in older adults, while amyloid-PET following tau-PET yields more limited gains. This pattern aligns with findings from Smith et al. [31], who showed that tau-PET improves diagnostic certainty even after extensive clinical workup, especially in amyloid-positive individuals, consistent with our observation of the highest PPV and NPV in this group.

In the context of the included TAP-TAU cases [18], a direct comparison between modeled PPV/NPV values and changes in clinician-estimated diagnostic certainty after tau-PET is not possible. However, examining how both are modulated by a tau-PET result can still be informative, e.g., by guiding the decision of whether to perform a scan in a given patient. In the tau-PET positive case, modeled and clinician-based probabilities were highly similar (90% vs. 95%). In the tau-PET negative case, the divergence was also small (19% vs. 10%), underscoring how a negative scan can meaningfully lower a clinician’s suspicion of AD and redirect attention toward alternative etiologies, even when AD initially seemed likely.

A key strength of the present study is the integration of sensitivity and specificity estimates from multiple autopsy-validated cohorts, reducing bias associated with single-cohort analyses. This approach, however, also introduced heterogeneity, as included studies differed in PET-to-autopsy intervals and ROIs used [9, 13, 20, 24]. Notably, while most studies derived tau-PET positivity thresholds using Youden’s index to optimally separate Braak V/VI from lower Braak stage pathology, one study [13] calibrated its threshold to distinguish visually positive from visually negative scans, which may have slightly influenced the pooled specificity estimate. Furthermore, not all studies used the exact temporal meta-ROI, potentially influencing the pooled performance metrics for identifying Braak V/VI pathology. Several other limitations should be noted. First, our analyses focused exclusively on [^18^F]flortaucipir, given that this is currently the only tau tracer with sufficient autopsy-validated data to derive reliable sensitivity and specificity estimates for Braak V/VI pathology using histology. While tau-PET positivity rates appear broadly similar across tracers [24], differences in binding characteristics may still affect PPV/NPV estimates. Second, we modelled tau-PET as a binary outcome, whereas differences in uptake magnitude between individuals likely still convey important additional diagnostic information. Third, since we explicitly focused on Braak V/VI pathology in our definition of clinicopathological AD, patients with cognitive impairment driven by earlier-stage tau pathology (Braak III/IV) will likely test tau-PET negative and be classified as true negatives in our framework, even though their impairment may be due to AD. This may lead to some overestimation of NPV, though its magnitude is likely modest given that symptomatic AD at Braak III/IV, while documented, is considerably less common than at Braak V/VI [32, 33]. Thus, our choice reflects both an intrinsic property of [^18^F]flortaucipir, which mainly captures advanced neocortical tau pathology (i.e., Braak V/VI) at autopsy [9, 13, 20, 24, 34], and the deliberate anchoring to Braak V/VI, motivated by the strong association with cognitive impairment [32, 35, 36]. Fourth, the CU tau-PET positivity rates used in our model capture a heterogeneous mix of individuals, including true preclinical AD cases and individuals without AD pathology who test positive, for example due to excessive non-specific binding. Their net directional effect on our PPV and NPV estimates cannot be fully determined, though the heterogeneity arguably reflects the clinical reality encountered in memory clinics. Fifth, the PET-to-autopsy intervals across included studies ranged from approximately 3 months to 4.2 years, which may introduce some bias in the pooled sensitivity and specificity estimates, an inherent limitation of multi-cohort autopsy studies. Additionally, in older patients with mixed pathologies, a positive tau-PET scan may reflect co-existing Braak V/VI pathology that is not the primary driver of cognitive symptoms, potentially overestimating PPV in this subgroup. This challenge is not unique to tau-PET but applies broadly to biomarker-based diagnosis in the context of mixed pathology. Notably, the age-related decline in PPV observed in our results partly reflects this phenomenon, as the model incorporates increasing tau-PET positivity rates in cognitively unimpaired older individuals to account for the rising prevalence of tau-PET positivity unrelated to clinicopathological AD as we defined it in this work. Finally, PPV and NPV estimates relied on clinician-estimated pre-PET AD probabilities, which are inherently subjective and context-dependent; nevertheless, we argue that incorporating them remains essential, as they reflect real-world diagnostic knowledge that can add information beyond data-derived metrics [37, 38].

In conclusion, [^18^F]flortaucipir tau-PET provides substantial diagnostic value for identifying clinicopathological AD, with its utility depending on age, pre-PET AD probability, and amyloid status. By explicitly modeling these factors, we present a probabilistic framework that allows clinicians to anticipate the expected gain in diagnostic confidence before ordering a tau-PET scan, supporting more informed and individualized imaging decisions in routine clinical practice. 

## Supplementary Information

Supplementary Material 1.

## Data Availability

Data and code are available on GitHub (OssenKoppeLab/BvanTol_TauPET_PPV_NPV_AD probability) and from the corresponding author upon reasonable request.
