# Supplementary material for: A probabilistic framework for clinicopathological Alzheimer’s disease using tau-PET
Source: Alzheimers Res Ther. 2026 Jul 11;18:223. doi: 10.1186/s13195-026-02133-1 (PMC13647783; doi:10.1186/s13195-026-02133-1)
Supplement: Supplementary file 1 [file 13195_2026_2133_MOESM1_ESM.docx]

**Supplementary Table 1** Overview of thresholding strategies, sensitivity, and specificity of [¹⁸F]flortaucipir PET for Braak V/VI NFT pathology across included studies

| **Study** | **Size (N)** |  | **PET ROI** | **PET Thresholding Method** | **PET Threshold** | **SEN (%)** | **SPE (%)** |
| --- | --- | --- | --- | --- | --- | --- | --- |
| Josephs et al.[1] | 248 |  | Inferior temporal | Youden-derived index for distinguishing BrV/VI from Br<V | 1.48 | 97.1 | 78.0 |
| Josephs et al.[1] | 248 |  | Superior parietal | Youden-derived index for distinguishing BrV/VI from Br<V | 1.23 | 90.0 | 67.0 |
| Vrillon et al.[2] | 71 |  | Temporal meta | SUVR > mean + 2 SD above cognitively normal controls | 1.35 | 95.0 | 100 |
| Fleisher et al.[3] | 60 |  | MUBADA Neocortex | Youden-derived index for distinguishing positive vs negative PET (visual read) | 1.113 | 84.2 | 100 |
| Soleimani-Meigooni et al.[4] | 20 |  | Braak V/VI ROI | SUVR > mean + 2 SD above cognitively normal controls | Not reported (~1.25 from figure) | 100 | 100 |
| ADNI (Coomans et al.[5]) | 12 |  | Temporal meta | Youden-derived index for distinguishing BrV/VI from Br<V | 1.29 | 75.0 | 87.5 |
| ADNI (Coomans et al.[5]) | 11 |  | Whole brain ROI | Youden-derived index for distinguishing BrV/VI from Br<V | 1.17 | 50.0 | 87.5 |
| ADC (Coomans et al.[5])^a^ | 9 |  | Temporal meta | Youden-derived index for distinguishing Br5-6 from Br<5 | 1.31 – 1.67 | 66.7 | 100 |
| ADC (Coomans et al.[5])^a^ | 9 |  | Whole brain ROI | Youden-derived index for distinguishing BrV/VI from Br<V | 1.20 – 1.69 | 66.7 | 100 |

^a^ For the ADC cohort, SUVR thresholds are reported as ranges rather than single values. As illustrated in Supplementary Figure 1, no data points fall within these intervals, meaning any threshold selected within the reported range yields identical sensitivity and specificity for detecting Braak V/VI NFT pathology. The range therefore reflects the resolution of the underlying data; given the small sample size, no single optimal cutoff could be identified.

**Supplementary Table 2** Overview of included studies reporting *whole brain* tau-PET sensitivity and specificity for Braak V/VI NFT pathology

| **Study** | **Sample size (N)** | **ROI used** | TP | FN | TN | **FP** | **SEN (%)** | **SPE (%)** |
| --- | --- | --- | --- | --- | --- | --- | --- | --- |
| Josephs et al.[1] | 248 | Superior Parietal | 93 | 10 | 97 | 48 | 90.0 | 67.0 |
| Fleisher et al.[3] | 60 | MUBADA neocortex | 32 | 6 | 22 | 0 | 84.2 | 100 |
| Soleimani-Meigooni et al.[4] | 20 | Braak V/VI ROI | 8 | 0 | 12 | 0 | 100 | 100 |
| ADNI (Coomans et al.[5]) | 11 | Whole brain ROI | 2 | 2 | 6 | 1 | 50.0 | 87.5 |
| ADC (Coomans et al.[5]) | 9 | Whole brain ROI | 4 | 2 | 3 | 0 | 66.7 | 100 |
| Pooled | 349 |  | 139 | 20 | 140 | 49 | 87.4 | 74.1 |

**Supplementary Table 3** Age-dependent prevalence rates of tau-PET positivity in cognitively unimpaired individuals (adapted from Ossenkoppele et al.[6])

| **Reported Age** | **Reported Prevalence (%)** | **Adapted Age Range** | **Adapted Prevalence (%)** |
| --- | --- | --- | --- |
| 50 | 2.4 | 50-55 | 2.8 |
| 55 | 3.2 | 55-60 | 3.7 |
| 60 | 4.2 | 60-65 | 4.9 |
| 65 | 5.6 | 65-70 | 6.5 |
| 70 | 7.4 | 70-75 | 8.6 |
| 75 | 9.8 | 75-80 | 11.3 |
| 80 | 12.8 | 80-85 | 14.7 |
| 85 | 16.5 | 85-90 | 18.8 |
| 90 | 21.1 |  |  |

Reported prevalence are generalized estimating equation (GEE)-based point estimates of tau-PET positivity from Ossenkoppele et al.[6] For the present study, we converted these to 5-year age ranges (i.e. 50-55, 55-60, etc.) by averaging the two bounding estimates (e.g., the prevalence for age range 50-55 is the mean of the prevalence estimates at ages 50 and 55). These adapted values were then used in PPV/NPV modeling.

**Supplementary Table 4** Definitions of terms used in the PPV and NPV formulas

| Term | Definition |
| --- | --- |
| P_AD, pre_ | (Hypothetical) clinician-estimated pre-PET probability of AD being the primary etiology driving cognitive impairment. |
| Se_tauPET_ | Proportion of cases with Braak V/VI NFT pathology at autopsy correctly identified as positive by tau-PET. |
| Sp_tauPET_ | Proportion of cases without Braak V/VI NFT pathology at autopsy correctly identified as negative by tau-PET. |
| P_tauPET+,CU_ | Prevalence of tau-PET positivity in cognitively unimpaired individuals at a given age. |
| True Positives | Proportion of cases with a positive tau-PET scan among individuals who have clinicopathological AD. |
| True Negatives | Proportion of cases with a negative tau-PET scan among individuals who do not have clinicopathological AD. |
| False Negatives | Proportion of cases with a negative tau-PET scan among individuals who do have clinicopathological AD. |
| FalsePositives_pathological_ | Proportion of cases with a positive tau-PET scan among individuals without Braak V/VI NFT pathology at autopsy – termed *pathological false positives.* This group is estimated by a probabilistically multiplying three factors: the pre-PET probability that an individual does not have AD (1 - P_AD, pre_), the proportion of non-AD individuals who are tau-PET negative (1 - P_tauPET+,CU_), and the probability that tau-PET nonetheless incorrectly indicates such negative cases as positive (1 - Sp_tauPET_). |
| FalsePositives_clinicopathological_ | Proportion of cases with a positive tau-PET scan and Braak V/VI NFT pathology at autopsy, but whose cognitive impairment is not primarily driven by AD – termed *clinicopathological false positives*. This group is estimated by multiplying three factors: the pre-PET probability that an individual does not have AD (1 - P_AD, pre_), the probability that tau-PET correctly detects Braak V/VI pathology (Se_tauPET_), and the age-dependent probability that CU individuals show tau-PET positivity that reflects NFT pathology (P_tauPET+,CU_). |

**Supplementary Table 5** Temporal tau-PET PPV with 95% confidence intervals

| **Age & P_AD,pre_** | **50-55** | **55-60** | **60-65** | **65-70** | **70-75** | **75-80** | **80-85** | **85-90** |
| --- | --- | --- | --- | --- | --- | --- | --- | --- |
| **0.9** | 0.98  [0.97-0.98] | 0.98  [0.97-0.98] | 0.98  [0.97-0.98] | 0.98  [0.97-0.98] | 0.97  [0.97-0.98] | 0.97  [0.97-0.98] | 0.97  [0.96-0.97] | 0.97  [0.96-0.97] |
| **0.8** | 0.95  [0.94-0.97] | 0.95  [0.94-0.96] | 0.95  [0.94-0.96] | 0.95  [0.94-0.96] | 0.94  [0.93-0.95] | 0.94  [0.93-0.95] | 0.93  [0.92-0.94] | 0.92  [0.92-0.93] |
| **0.7** | 0.92  [0.90-0.94] | 0.92  [0.90-0.94] | 0.92  [0.90-0.94] | 0.91  [0.89-0.93] | 0.91  [0.89-0.92] | 0.90  [0.88-0.91] | 0.89  [0.87-0.90] | 0.88  [0.86-0.89] |
| **0.6** | 0.89  [0.86-0.91] | 0.88  [0.85-0.91] | 0.88  [0.85-0.90] | 0.87  [0.84-0.89] | 0.86  [0.84-0.89] | 0.85  [0.83-0.87] | 0.84  [0.82-0.86] | 0.82  [0.80-0.84] |
| **0.5** | 0.84  [0.80-0.87] | 0.83  [0.80-0.87] | 0.83  [0.79-0.86] | 0.82  [0.78-0.85] | 0.81  [0.77-0.84] | 0.79  [0.76-0.82] | 0.77  [0.75-0.80] | 0.75  [0.73-0.78] |
| **0.4** | 0.77  [0.73-0.82] | 0.77  [0.72-0.81] | 0.76  [0.72-0.80] | 0.75  [0.71-0.79] | 0.73  [0.70-0.77] | 0.72  [0.68-0.75] | 0.70  [0.66-0.73] | 0.67  [0.64-0.70] |
| **0.3** | 0.69  [0.63-0.75] | 0.68  [0.63-0.74] | 0.67  [0.62-0.73] | 0.66  [0.61-0.71] | 0.64  [0.59-0.69] | 0.62  [0.58-0.66] | 0.59  [0.56-0.63] | 0.57  [0.54-0.60] |
| **0.2** | 0.56  [0.50-0.64] | 0.55  [0.50-0.62] | 0.54  [0.49-0.61] | 0.53  [0.48-0.59] | 0.51  [0.46-0.56] | 0.49  [0.44-0.53] | 0.46  [0.42-0.50] | 0.43  [0.40-0.47] |
| **0.1** | 0.36  [0.31-0.44] | 0.36  [0.30-0.42] | 0.34  [0.30-0.41] | 0.33  [0.29-0.39] | 0.31  [0.28-0.36] | 0.30  [0.26-0.34] | 0.28  [0.25-0.31] | 0.25  [0.23-0.28] |

Values represent the pooled point estimate of PPV [95% confidence interval]. The point estimate is computed directly as ΣTP/(ΣTP+ΣFN) and ΣTN/(ΣTN+ΣFP) across all five studies. To derive uncertainty estimates, 2×2 contingency tables (TP, FP, TN, FN) from the five included autopsy-validated studies were resampled with replacement across 5,000 iterations. For each iteration, pooled sensitivity and specificity were recomputed as ΣTP/(ΣTP+ΣFN) and ΣTN/(ΣTN+ΣFP) across all studies and propagated through the PPV and NPV formulas. The 2.5th and 97.5th percentiles of the resulting distribution define the 95% confidence interval for each cell.

**Supplementary Table 6** Temporal tau-PET NPV with 95% confidence intervals

| **Age & P_AD,pre_** | **50-55** | **55-60** | **60-65** | **65-70** | **70-75** | **75-80** | **80-85** | **85-90** |
| --- | --- | --- | --- | --- | --- | --- | --- | --- |
| **0.1** | 0.99  [0.99-0.99] | 0.99  [0.98-0.99] | 0.99  [0.98-0.99] | 0.99  [0.98-0.99] | 0.99  [0.98-0.99] | 0.99  [0.98-0.99] | 0.99  [0.98-0.99] | 0.99  [0.98-0.99] |
| **0.2** | 0.98  [0.97-0.99] | 0.98  [0.97-0.99] | 0.98  [0.97-0.99] | 0.98  [0.97-0.99] | 0.98  [0.96-0.99] | 0.98  [0.96-0.99] | 0.98  [0.96-0.99] | 0.97  [0.96-0.99] |
| **0.3** | 0.96  [0.94-0.98] | 0.96  [0.94-0.98] | 0.96  [0.94-0.98] | 0.96  [0.94-0.98] | 0.96  [0.94-0.98] | 0.96  [0.94-0.98] | 0.96  [0.94-0.98] | 0.96  [0.93-0.98] |
| **0.4** | 0.94  [0.92-0.97] | 0.94  [0.92-0.97] | 0.94  [0.91-0.97] | 0.94  [0.91-0.97] | 0.94  [0.91-0.97] | 0.94  [0.91-0.97] | 0.94  [0.91-0.96] | 0.93  [0.90-0.96] |
| **0.5** | 0.92  [0.88-0.95] | 0.92  [0.88-0.95] | 0.92  [0.88-0.95] | 0.91  [0.88-0.95] | 0.91  [0.87-0.95] | 0.91  [0.87-0.95] | 0.91  [0.87-0.95] | 0.90  [0.86-0.94] |
| **0.6** | 0.88  [0.83-0.93] | 0.88  [0.83-0.93] | 0.88  [0.83-0.93] | 0.88  [0.82-0.93] | 0.88  [0.82-0.93] | 0.87  [0.82-0.93] | 0.87  [0.81-0.92] | 0.86  [0.80-0.92] |
| **0.7** | 0.83  [0.76-0.90] | 0.83  [0.76-0.90] | 0.82  [0.75-0.89] | 0.82  [0.75-0.89] | 0.82  [0.75-0.89] | 0.81  [0.74-0.89] | 0.81  [0.73-0.88] | 0.80  [0.72-0.88] |
| **0.8** | 0.74  [0.65-0.84] | 0.73  [0.64-0.83] | 0.73  [0.64-0.83] | 0.73  [0.64-0.83] | 0.72  [0.63-0.83] | 0.72  [0.63-0.82] | 0.71  [0.62-0.82] | 0.70  [0.60-0.81] |
| **0.9** | 0.55  [0.45-0.69] | 0.55  [0.45-0.69] | 0.55  [0.44-0.69] | 0.54  [0.44-0.68] | 0.54  [0.43-0.68] | 0.53  [0.43-0.67] | 0.52  [0.42-0.66] | 0.51  [0.40-0.65] |

**Supplementary Table 7** Age-dependent prevalence rates of amyloid-PET positivity in CU individuals (adapted from Janssen et al.[7])

| **Reported Age** | **Reported Prevalence (%)** | **Adapted Age Range** | **Adapted Prevalence (%)** |
| --- | --- | --- | --- |
| 50 | 12.3 | 50-55 | 13.6 |
| 55 | 14.8 | 55-60 | 16.2 |
| 60 | 17.6 | 60-65 | 19.3 |
| 65 | 20.9 | 65-70 | 22.8 |
| 70 | 24.6 | 70-75 | 26.7 |
| 75 | 28.7 | 75-80 | 31.0 |
| 80 | 33.2 | 80-85 | 35.6 |
| 85 | 38.0 | 85-90 | 40.6 |
| 90 | 43.1 |  |  |

Reported values are generalized estimating equation (GEE)-based point estimates of amyloid-PET positivity from Janssen et al.[7] These were converted to 5-year age ranges by averaging the two bounding prevalence estimates, following the same procedure as for tau-PET (**Supplementary Table-3**), and used in PPV/NPV modelling.

**Supplementary Figure 1** Scatterplots of [^18^F]flortaucipir SUVR values by Braak stage in the ADC autopsy cohort

(**A)** Temporal meta-ROI SUVR values. (**B**) Whole-brain ROI SUVR values. The red-shaded bands indicate the range of SUVR thresholds yielding the same sensitivity and specificity for detecting Braak V–VI NFT pathology. Participant inclusion criteria[5] were: age ≥50 years, availability of a tau-PET scan suitable for regional SUVR estimation, and autopsy with Braak NFT staging. In the ADC cohort (N = 9), mean age was 71.1 years and 55.6% were female.

**Supplementary Figure 2** Scatterplots of [^18^F]flortaucipir SUVR values by Braak stage in the ADNI autopsy cohort

(**A**) Temporal meta-ROI SUVR values. **(B)** Whole-brain ROI SUVR values. The red line indicates the Youden-derived SUVR threshold for detecting Braak V–VI NFT pathology. In the ADNI cohort (N = 12), mean age was 82.5 years and 25% were female[5].

**Supplementary Figure 3** Temporal tau-PET PPV and NPV tables stratified by APOE ε4 genotype

**Supplementary Figure 4** Whole brain tau-PET PPV and NPV

**Supplementary Figure 5** Combined PPV and NPV after whole brain amyloid-PET and subsequent tau-PET

**Supplementary Figure 6** Combined PPV and NPV after whole brain tau-PET and subsequent amyloid-PET

**References**

1.	Josephs KA, Tosakulwong N, Weigand SD, Graff-Radford J, Schwarz CG, Senjem ML, et al. Flortaucipir PET uncovers relationships between tau and amyloid-β in primary age–related tauopathy and Alzheimer’s disease. Science Translational Mediciner. 2024;16(eado8076).

2.	Vrillon A, Spina S, Mejia-Perez J, Lamore T, Yballa C, Soleimani-Meigooni DN, et al. Association of [(18)F]Flortaucipir-PET and plasma p-tau217 with tau neuropathology in AD and other neurodegenerative disorders. medRxiv. 2025.

3.	Fleisher AS, Pontecorvo MJ, Devous MD, Sr., Lu M, Arora AK, Truocchio SP, et al. Positron Emission Tomography Imaging With [18F]flortaucipir and Postmortem Assessment of Alzheimer Disease Neuropathologic Changes. JAMA Neurol. 2020;77(7):829–39.

4.	Soleimani-Meigooni DN, Iaccarino L, La Joie R, Baker S, Bourakova V, Boxer AL, et al. 18F-flortaucipir PET to autopsy comparisons in Alzheimer's disease and other neurodegenerative diseases. Brain. 2020;143(11):3477–94.

5.	Coomans EM, van Tol B, Groot C, Smith R, Palmqvist S, Stomrud E, et al. Quantitation of PET spatial extent as a potential adjunct to visual interpretation of [(18)F]flortaucipir imaging: TAU-SPEX. Eur J Nucl Med Mol Imaging. 2025.

6.	Ossenkoppele R, Coomans EM, Apostolova LG, Baker SL, Barthel H, Beach TG, et al. Tau PET positivity in individuals with and without cognitive impairment varies with age, amyloid-beta status, APOE genotype and sex. Nat Neurosci. 2025.

7.	Jansen WJ, Janssen O, Tijms BM, Vos SJB, Ossenkoppele R, Visser PJ, et al. Prevalence Estimates of Amyloid Abnormality Across the Alzheimer Disease Clinical Spectrum. JAMA Neurol. 2022;79(3):228–43.
